# Back to basics: The effects of block vs. interleaved trial administration on pro- and anti-saccade performance

**DOI:** 10.1371/journal.pone.0172485

**Published:** 2017-02-21

**Authors:** Liran Zeligman, Ari Z. Zivotofsky

**Affiliations:** 1 Dept. of Psychology, Bar Ilan University, Ramat Gan, Israel; 2 The Leslie and Susan Gonda (Goldschmied) Multidisciplinary Brain Research Center, Bar-Ilan University, Ramat Gan, Israel; Centre de neuroscience cognitive, FRANCE

## Abstract

The pro and anti-saccade task (PAT) is a widely used tool in the study of overt and covert attention with promising potential role in neurocognitive and psychiatric assessment. However, specific PAT protocols can vary significantly between labs, potentially resulting in large variations in findings across studies. In light of recent calls towards a standardization of PAT the current study's objective was to systematically and purposely evaluate the effects of block vs. interleaved administration—a fundamental consideration—on PAT measures in a within subject design. Additionally, this study evaluated whether measures of a Posner-type cueing paradigm parallels measures of the PAT paradigm. As hypothesized, results indicate that PAT performance is highly susceptible to administration mode. Interleaved mode resulted in larger error rates not only for anti (blocks: M = 22%; interleaved: M = 42%) but also for pro-saccades (blocks: M = 5%; interleaved: M = 12%). This difference between block and interleaved administration was significantly larger in anti-saccades compared to pro-saccades and cannot be attributed to a 'speed/accuracy tradeoff'. Interleaved mode produced larger pro and anti-saccade differences in error rates while block administration produced larger latency differences. Results question the reflexive nature of pro-saccades, suggesting they are not purely reflexive. These results were further discussed and compared to previous studies that included within subject data of blocks and interleaved trials.

## Introduction

In the pro and anti-saccade task [1] (herein: PAT) participants are required to generate a saccade towards a specific location in space (pro-saccade) or to the opposite direction (anti-saccade). Thus, like its antecedent cueing paradigm [2], it is a powerful tool in the study of overt vs. covert attention (for a review: [3–4]). By 'overt' attention is meant the allocation of attention via eye gaze (i.e. fixating the fovea on the attendant space), whereas 'covert' attention is the allocating of attention without a concurrent gaze shift.

Typically, pro-saccades are stimulus driven, difficult to inhibit, reflexive shifts of gaze. Anti-saccades are typically goal driven and require volitional, "top down" processing such as inhibiting the potent tendency to generate a reflexive, pro-saccade towards the target. Overall, subjects typically report anti-saccades as "harder" to execute, while exhibiting slower latencies for correct anti-saccades and more errors than in pro-saccades [3–4]. These differences, often referred to as the "anti-saccade cost," have been widely reported in previous research [1], [5–6] and are used to measure overt vs. covert attention shifts [7].

The executive function associated with anti-saccade generation has also made it a potentially useful tool in neurocognitive assessment (for a review: [8]). This has been widely studied among people with schizophrenia [9–10], consistently showing higher anti-saccade errors compared to controls [8]. Other disorders have also showed anti-saccade deficits and may share a possible common feature of prefrontal dysfunction. These include ADHD [11–12], OCD [13–14] and prefrontal lesions (for a review: [15]).

There is, however one major pitfall: While all variants of the PAT share the requirement of reflexive and volitional shifts of overt and covert attention, and as such has been generally assumed to be functionally equivalent [8], there are many methodological parameters on which PAT can differ. For example, different variations of timing, number of targets, block vs interleaved administration, exogenous vs endogenous stimuli, and peripheral vs central cueing to generate pro or anti-saccades, are some of them. Considering the fact that even task instruction has been proven to affect saccadic tracking [16] and saccadic error rates [17] these parameters can have a considerable impact on saccadic outcome measures and must be taken into account when interpreting PAT data.

Understanding that each laboratory adopts its own unique methodological procedure, together with the potential impact test administration has on the resultant data, suggests that it is possible that different labs will produce divergent results regardless of experimental manipulation or other relevant variables [18]. As a result, there are large variations in findings across studies. For example, Evdokimidis and colleagues [19] sampled 2006 subjects and reported an average anti-saccade latency of 270ms, while Derakshan and colleagues [20] found a much higher average latency of 344ms in 61 subjects. Arguably, this difference might be attributable to the inclusion of a secondary Posner-like recognition task in Derakshan's study. Error rates too vary extensively: Smyrnis [21] reports a range of 2% to 30%, but much higher error rates have also been observed, for example 43% (e.g. [22], Exp 1c), making it rather hard to draw definitive conclusions upon variations in specific saccade performance.

Another noteworthy point is that while conclusions are made for what seems to be the same theoretical overt and covert attention processes, each study uses somewhat different methodology and analysis- without questioning whether that specific protocol truly represents the same parameter. For instance, Posner's concept of covert attention is measured quite differently than PAT's covert attention measures: Posner used recognition RT's while PAT utilize saccadic latency and error rates. These discrepancies also exist, though much more subtly, within different variations of the PAT.

A good example is the case of central vs peripheral trial cueing. In an interleaved administration of PAT, participants receive different cues that instruct them as to the generation of either a pro or anti-saccade. The command is usually a change of a specific attribute such as color or shape of the cue (e.g. green for pro-saccade and red for anti). Typically, in central cueing the central fixation symbol is used to both fixate and to instruct the saccade type (used e.g. by Derakshan [20]). In peripheral cueing it is the peripheral target which serves a dual function of also instructing the saccade type to be executed (used e.g. by [23]). The latter requires the subject to first covertly shift attention to the peripheral target in order to receive the instruction whether a pro or anti-saccade should be generated. This is of course very different when compared to central cueing where attention need not be shifted at all in order to determine the saccade type.

In the above example, two studies which used different methodologies, and thus utilized different process of attention shifting, will both eventually draw conclusions on what seems to be same theoretical constructs of overt and covert attention. Indeed, covert attention to a peripheral cue seems to have slower latencies compared to central cueing. Chiau et al. [24], for example, used peripheral cueing and reported slower latencies (anti 470 ms; pro 460ms) than Derakshan, et al. [20] who used central cueing (anti 344 ms pro 238ms).

These difficulties have led to recent attempts towards a standardized protocol and analysis of PAT [25]. Much of their suggestions relied on their extensive personal experiences. For instance, in the matter of block vs interleaved design Antoniades and colleagues suggested using a set of 5 blocks: starting with 60 pro-saccades trials, then three blocks of 40 anti-saccades each and ending with another block of 60 pro-saccades. The authors felt that this procedure provides a reasonable tradeoff between number of trials and participant fatigue. They did not, however, elaborate on what rationale led them to suggest blocks over interleaved administration, nor on the possible outcomes this particular choice might have on saccadic latency and error rates. In order to seriously consider using these tests for clinical assessment, one has to go back to basics and to scientifically evaluate the effects test characteristic have on saccadic performance.

The core choice of block vs interleaved administration is a fundamental consideration in PAT design and has been shown to be relevant in other paradigms as well. For example, it is known that the detection of letters improves when "globally" processing real-word strings compared to "analytically" processing non-word strings [26]. In this paradigm it is crucially important whether the tasks are presented in blocks or interleaved, because the effect disappears when presenting trials in blocks as opposed to interleaved [27], thus, suggesting that methodological factors play a key role in global vs. analytical processing.

To the best of our knowledge, no previous study has set out as its primary research objective to test the effects of administration mode (block vs interleaved) in PAT. There are, however, a few studies that were not primarily interested in the effects of administration type per se, but did include some variation of both interleaved and block PAT. Olk and Kingstone [23] for example, examined 6 subjects in a peripheral-trial-cue PAT. They found that interleaved administration increased antisaccade error rates and both pro and anti-saccade latency. As a result, pro and anti-saccade latency differences decreased in interleaved mode, while error rate differences actually increased. This pattern was not replicated in their second experiment when an additional irrelevant target was added. Their use of peripheral cueing makes it difficult to draw any conclusions on overt and covert attention differences. Is it true that interleaved mode eliminates antisaccade cost? Or is it limited to peripheral cueing?

While there are a few other studies which, *inter alia*, included both block and interleaved conditions, and from which some information regarding the difference can be gleaned [28–30], no study to date has set out to specifically and explicitly investigate the effects of administration mode (blocks vs interleaved) on PAT measures. It is vitally important that researchers and clinicians from the psychiatric and neurocognitive communities have such a study for otherwise some will be comparing apples and oranges.

The objective of the current study was to investigate the effects of administration mode (block vs interleaved) on saccadic errors and latencies, within subjects and with all other parameters held fixed. In addition, we were also interested to see whether Posner-like recognition task measures correspond to PAT measures of overt and covert attention. In the discussion, we will compare our results to some of the previous studies which included within subject data of blocks and interleaved trials.

Our primary hypothesis is that administration method matters. We predict that trials run as blocks will yield different results than those run interleaved, even when performed in the same lab with all other factors and parameters held fixed. More specifically, we hypothesize that interleaved administration will increase directional error rates for anti but not for pro-saccades. However, anti-saccade latency is not hypothesized to be affected as they are (relatively) quite slow to begin with.

## Methods

### Participants

The participants were 21 right-handed undergraduate students between the ages of 20 and 22 (7 males) who were recruited via convenient sampling on the campus of Bar-Ilan University (BIU). All participants had normal or corrected to normal vision, and were healthy with no known neurological or psychological conditions. The experiment was approved by the Bar Ilan University human subjects committee, and all subjects gave written informed consent prior to participation. The work described herein was carried out in accordance with the Code of Ethics of the World Medical Association (Declaration of Helsinki).

### Set up and eye movement recordings

Participants sat in a dimly lit room in front of a 19'' CRT monitor (LG Flatron F900p) on which the stimulus was displayed. An adjustable chin & forehead rest was used in order to minimize head movements and to ensure a constant distance between the eyes and screen (76 cm). Monocular eye movements were recorded with the ISCAN ETL-400 (ISCAN Inc., Woburn, MA) video-based eye tracker at 120 Hz sampling rate using a 5 point calibration. The ISCAN ETL-400 uses a video-based, dark pupil-to-cornea reflection method. Differences between the corneal Purkinje image 1 and pupil center are calculated as changes in eye position.

### General procedure and design

The current study combined a PAT and a Posner-like discrimination task. To do so, we adapted and modified a target-target cueing paradigm design (e.g. [31]) in which participants responded to the first target with a saccade and to the second target with a manual (key press) response. All participants completed the "Blocks" and "Interleaved" administration modes. In the Block condition, pro- and anti- saccades were administered in two separate blocks. The order of blocks (pro first or anti first) was counterbalanced across participants. In the Interleaved condition, pro and anti- saccades were randomly interleaved.

**Fig 1:**
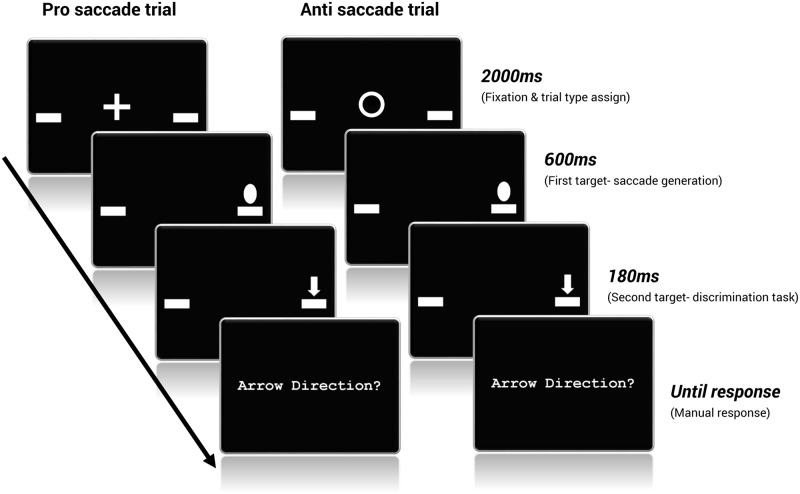
Example of typical pro- and anti- saccade trial sequence. On the left, fixation instructs a generation of a pro-saccade (pro trial), first target appears on the right- a saccade to the right would be a "correct" response. Second target appears at the same (cued) location as first target, pressing the keyboard "up arrow" quickly would be a correct response. On the right, fixation instructs a generation of anti-saccade (anti trial), a saccade to the left would be a correct response, the second target is at the same (cued) location of the first target. The figure is not drawn to scale.

Each trial began with a 2000 ms fixation at the center of the screen (see Fig 1) followed by the first target (an oval shape), which appeared for 600 ms (randomly and with equal probability) 10 degrees left or right of fixation. In interleaved trials fixation also served to indicate the saccade type: a plus sign for pro and a circle for anti. For pro trials participants were instructed to look as fast as possible towards the flashed peripheral target. For anti trials they were instructed to look as fast as possible to the opposite direction of the flashed target. Participants had 600 ms to generate a correct saccade. Immediately with target extinction, the second target appeared (an arrow) at either the left or right target position, randomly and with equal probability, for 180ms. Participants were asked to report whether the arrow was pointing up or down by pressing the corresponding keyboard button as fast and accurately as possible. The first target was not predictive of the second target location: on half of the trials the second target appeared at the same location (cued location) and on the remaining half at the opposite location (uncued location).

Saccadic accuracy and latency as a function of trial type (pro or anti) and administration mode (blocks or interleaved) were obtained from the first target. Manual recognition accuracy and response time as a function of trial type (Pro or Anti) and location (Cued or Uncued) were obtained from the second target.

This allows for a somewhat different dissociation of overt and covert attention. In successful pro-saccade trials, both overt and the covert attention is oriented towards the location of the first target. The second target may then appear, in the same (Cued) location attended by both overt and covert attention; or at the opposite (Uncued), unattended location. In contrast, a successful anti-saccade trial requires the orienting of covert attention towards the target, inhibiting a pro-saccade and the orienting of overt attention (anti-saccade) to the opposite direction. Thus, during the appearance of the second target attention is split, with covert attention allocated towards the cued location and overt attention towards the uncued location.

### Analysis

Analysis was performed off line using an Excel Macro. The software first examined the gaze location of the subject in the 20 ms prior to target appearance. If gaze did not fall within a predetermined radius of the center of the screen the trial was labeled as "fixation failure" and removed from further analysis.

The program next identified the first saccade following target appearance.

Saccades with latencies shorter than 100ms (0.3%) were excluded from analysis. These saccades, whether "express" [32] or "anticipatory" [33], are disputably thought to represent a qualitatively different type of saccades [4]. Saccades in the correct direction made within 600 ms of target appearance were labeled as "correct". A lack of eye movements or a generation of a saccade in the wrong direction was labeled as "error". Saccade latency used in the subsequent analysis was the time from target appearance to saccadic onset for correct saccades. Manual reaction times for correct responses of the arrow direction that were greater than 2SDs from the individual's mean were not included in the analysis. Cueing effect was calculated as Uncued *minus* Cued RT's. Positive values indicate facilitatory effects, and negative values indicate inhibition of return (IOR).

## Results

### Saccade error rates

**Fig 2:**
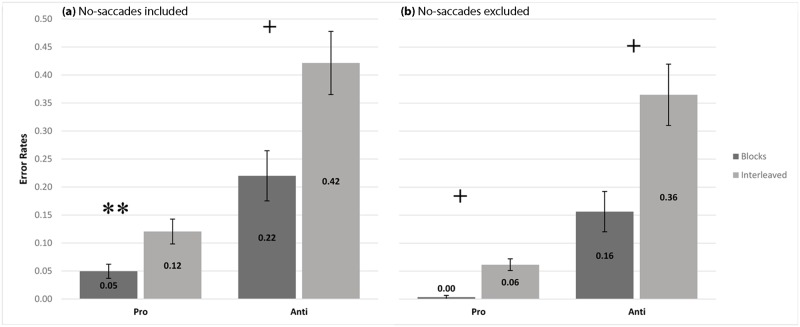
Mean saccade error rates for pro-saccade and anti-saccade trials under block and randomly assigned conditions. (a) Trials with no saccades included in analysis as errors. (b) Trials with no saccades excluded. Error bars represent the standard error of the mean. Simple effects were analyzed via Bonferroni post-hoc comparisons. **p<0.01, ^+^p<0.001.

We conducted a repeated measures ANOVA with administration (blocks/ interleaved) and trial type (pro-saccade/anti-saccade) as within subject independent variables. Analysis yielded a significant main effect for trial type *F*(1,20) = 26.34, *p*<0.0001, *η*^*2*^ = 0.56. Overall, participants made significantly fewer directional errors when asked to perform pro-saccades compared to anti-saccades (*M* = 0.08, *SD* = 0.06; *M* = 0.32, *SD* = 0.21). A main effect for administration was also significant *F*(1,20) = 38.94, *p*<0.0001, *η*^*2*^ = 0.66. Overall, participants made significantly fewer directional errors in the blocks compared to the interleaved administration (*M* = 0.13, *SD* = 0.11; *M* = 0.27, *SD* = 0.14). More interestingly, a significant interaction confirmed that the differences in error rates between blocks and interleaved administration becomes larger in anti-saccades compared to pro-saccades *F*(1,20) = 10.97, *p*<0.01, *η*^*2*^ = 0.35. Fig 2A shows the mean error rates in the Pro & Anti-saccade tasks as a function of test administration.

Next, we performed the same ANOVA excluding from analysis trials that lacked eye movements in the 600 ms time window. Results reproduced the same pattern- yielding significant main effects for trial type *F*(1,20) = 27.14, *p*<0.0001, *η*^*2*^ = 0.57, administration *F*(1,20) = 63.56, *p*<0.0001, *η*^*2*^ = 0.76 and interaction *F*(1,20) = 19.15, *p*<0.01, *η*^*2*^ = 0.48. Fig 2B shows mean error rates after excluding trials lacking eye movements.

### Saccade latencies

A 2(administration) by 2(trial type) repeated measure ANOVA yielded a significant main effect for trial type *F*(1,20) = 116.18, *p*<0.0001, *η*^*2*^ = 0.85. Overall, pro-saccades had shorter saccadic latencies compared to anti-saccades (*M* = 254, *SD* = 22; *M* = 326, *SD* = 31). There was no main effect for administration: overall, both randomized and blocked administration each had an average latency of 290ms (*M* = 290, *SD* = 24; *M* = 290, *SD* = 24). There was also a significant interaction *F*(1,20) = 10.07, *p*<0.01, *η*^*2*^ = 0.33. Bonferroni post hoc comparisons found no significant differences between blocks and randomized administration in either the pro or anti-saccades, suggesting that the source of the interaction can be attributed to the differences between pro and anti-saccades within each administration mode.

**Fig 3:**
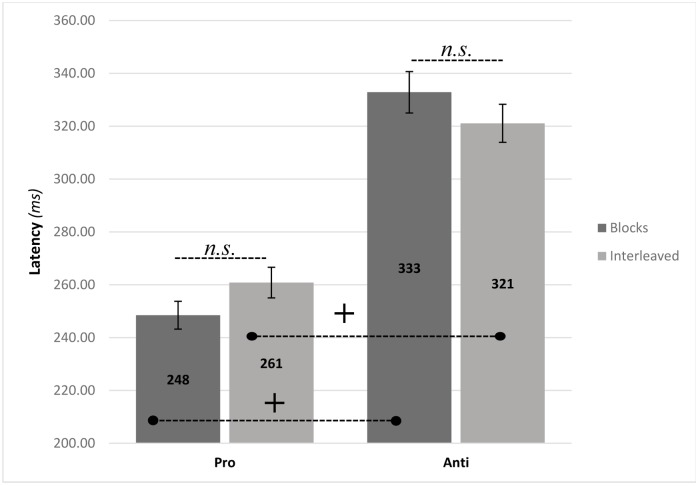
Mean latency (*ms*) of correct saccades for pro-saccade and anti-saccade trials under block and randomly assigned conditions. Error bars represent the standard error of the mean. Simple effects were analyzed via Bonferroni post-hoc comparisons. ^+^*p*<0.001.

**Fig 4:**
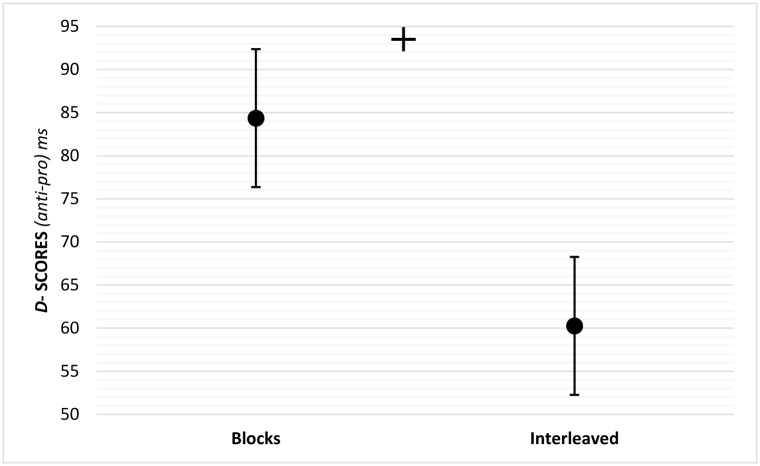
Mean *D*-scores calculated as *D = Anti-pro* latencies (*ms*) under block and interleaved conditions. Error bars represent the standard error of the mean. Dependent t-test was used for post-hoc comparison. ^+^*p*<0.001.

As is standardly done in the pro and anti tasks, we calculated the "anti-saccade cost" in randomized and in blocked administration as the differences between anti and pro-saccade latencies (*D = Anti-Pro*). Dependent samples t-test revealed that block administration results in larger anti-saccade cost (*M*_D-scores_ = 84, *SD* = 36) compared to randomized administration (*M*_D-scores_ = 60, *SD* = 33) *t*(20) = 3.174, *p*<0.01. Thus, the differences between pro and anti-saccade latencies are more pronounced when the test is administered in blocks compared to randomly interleaved. Fig 3 shows the mean latencies of correct pro and anti-saccades as a function of test administration, and Fig 4 shows latency *D* scores under blocked and randomized administration.

### Discrimination task error rates

Across all conditions, there were very few mistakes in recognizing the arrow's direction (Error rates ranging from a minimum of 1.2% to a maximum of 3.7%). This presumably indicates a floor effect. This was supported by the fact that although an administration*trial type interaction was significant *F*(1,20) = 10.14, *p* = 0.005, *η*^*2*^ = 0.33, simple effects analysis was not significant for any differences.

### Discrimination task reaction times (RTs)

**Fig 5:**
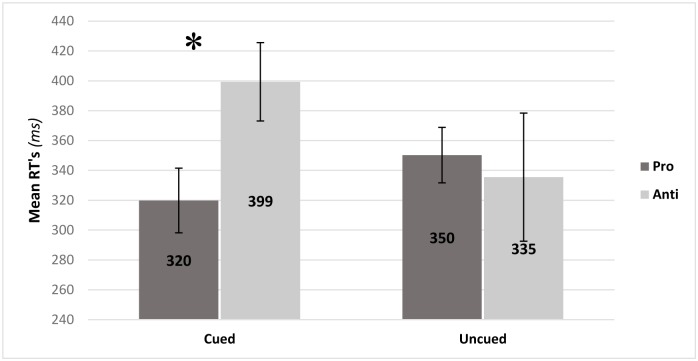
Mean correct recognition reaction times (ms) for pro-saccade and anti-saccade trials in cued and uncued conditions. Error bars represent the standard error of the mean. Simple effects were analyzed via Bonferroni post-hoc comparisons. *p<0.05.

Repeated measures ANOVA with administration (blocks/interleaved) trial type (pro/anti) and second target location (cued/uncued) as independent variables and correct recognition RT's (*ms*) yielded a significant location*trial type interaction *F*(1,20) = 4.41, *p* = 0.048, *η*^*2*^ = 0.18. As seen in Fig 5, anti-saccades had slower RT's than pro-saccade at the cued location. At the uncued location these differences were not significant. Additionally, the cueing effect (calculated as Uncued *minus* Cued RT's) for pro-saccades (M = +30.3) was significantly different from anti-saccades (M = -63.8). Positive values indicate facilitatory effects, and negative values indicate inhibition of return (IOR).

## Discussion

The current study's objectives was to evaluate the effects of block vs interleaved administration on PAT measures in a within subject design within the same laboratory and with all other variables held fixed. Additionally, we were also interested in evaluating the possible relationship between Posner-like manual recognition costs and PAT saccadic costs.

In agreement with many previous studies, the current data showed pro-saccades had lower error rates and shorter latencies compared to anti-saccades. These differences, often referred to as the "anti-saccade cost," are at the heart of the pro and anti-saccade task and have been widely reported in previous research [1], [6], [32]. This concept of an "anti-saccade cost" refers both to latencies and error rates, although the latency difference is usually more pronounced.

In accordance with our hypothesis, administration mode was found to play a significant role in PAT outcome measures. Interleaved mode resulted in larger error rates not only for anti (blocks: M = 22%; interleaved: M = 42%) but also for pro-saccades (blocks: M = 5%; interleaved: M = 12%). This difference between block and interleaved administration was significantly larger in anti-saccades compared to pro-saccades (Fig 2) and cannot be attributed to a 'speed/accuracy tradeoff'- as there were no significant latency differences between blocks and randomized trials (Fig 3). However, latency “anti-saccade cost” (D-scores) was larger in blocks administration (Fig 4). This was due to both the slowing, though not significantly, of the pro-saccades and the speeding up of the anti-, again not significantly, but together resulting in a significant decrease of anti-saccade cost in the interleaved mode.

These results can be attributed to a cognitive mechanism of proactive inhibitory control that has recently been suggested could explain pro-saccades RT differences between block and interleaved presentation [34–35]. Applying their logic to the present study leads to the following understanding: In block situations, the subjects are aware of the type of saccade they will be executing and thus in the pro trials are prepared to make a "reflexive" saccade while in anti-saccade blocks they have fully engaged proactive inhibitory control so as to suppress any reflexive saccades. That is, in the pro-saccade situation they are prepared to react instantly to the target, while in the anti-saccade situation they are prepared to not react to the target and avoid an automatic saccade towards the target. This leads to few errors and a large difference in reaction times between the two conditions. In the interleaved situation, the subjects do not know the trial type and, aware of the possibility of an anti-saccade, engage proactive inhibitory control to tamper the reflexive pro-saccades. On the other hand, there is a possibility that they will indeed need to make a pro-saccade and thus do not inhibit to the same degree as in blocks of anti-saccades. This intermediate state of inhibition leads to an elevated number of errors for both types of saccades. In addition, because pro-saccades are now somewhat restraint there is an increase in their RTs while a reduced level of inhibition leads to faster RTs for the correct anti-saccades. It should be noted that those studies [34–35] did not compare error rates in the various conditions and only studied pro-saccades in a variety of paradigms. This hypothesis could be tested by varying the ratio of pro to anti-saccades in a mixed condition. With a high percentage of anti-saccades it would be expected that greater inhibitory control would be exercised and thus RTs would be greater than in the present interleaved condition while decreasing the error rate for anti-saccades.

We hypothesized that interleaved administration will increase antisaccade error rates; surprisingly it also affected pro-saccades, which also showed an increase in error rates, although this increase was significantly smaller than the one found for anti-saccades. This finding has significant implications regarding our understanding of the nature of pro-saccades. Pro-saccades are widely considered to be reflexive, while anti-saccades are viewed as volitional [3–4]. The hard-to-inhibit express saccades observed in tests using a "gap" paradigm are often cited as evidence of the pure reflexive nature of pro-saccades and the affirmation of the visual-motor grasp reflex. If pro-saccades were indeed solely reflexive, then they should not be affected by test administration. Our results indicate that this is not the case; rather anti-saccades require more inhibition and are hence more, but not entirely, volitional while pro-saccades are less taxing on cognitive resources and hence are more, but not entirely, reflexive, as explained by the proactive inhibitory control theory.

This idea, that pro-saccades are not purely reflexive was previously suggested by Schall [36] who noted that the relatively long typical latency of pro-saccades indicates that they are not purely reflexive but are the culmination of a complex neural process. It is also consistent with the observed significant interaction by which the differences between blocks and randomized administration became more prominent when performing the "more volitional" anti-saccades. Similarly, increased cognitive load had an interfering effect on both pro-saccades and anti-saccades [37–38]; and, similar to our results, this interference effect was larger upon anti-saccades than pro-saccades [39].

The hindering of the pro-saccade advantage has been previously reported [23–24], although this was apparent in latency and not error rates. In addition, these studies used a peripheral trial-type-cue whereby the target, and not the fixation, was used to cue the execution of pro vs. anti-saccades. Thus, it could be argued that the slowing observed in those studies is due to the fact that in order to execute a saccade in this paradigm, one has to first covertly redirect attention to the peripheral target, decide between pro and anti-saccade, and only then generate and execute the desired saccade. Thus, it is not surprising that this pre-allocation of covert attention "handicaps" the pro-saccade "reflexive" advantage reported in classic experiments that use the central fixation to cue saccade type.

**Fig 6:**
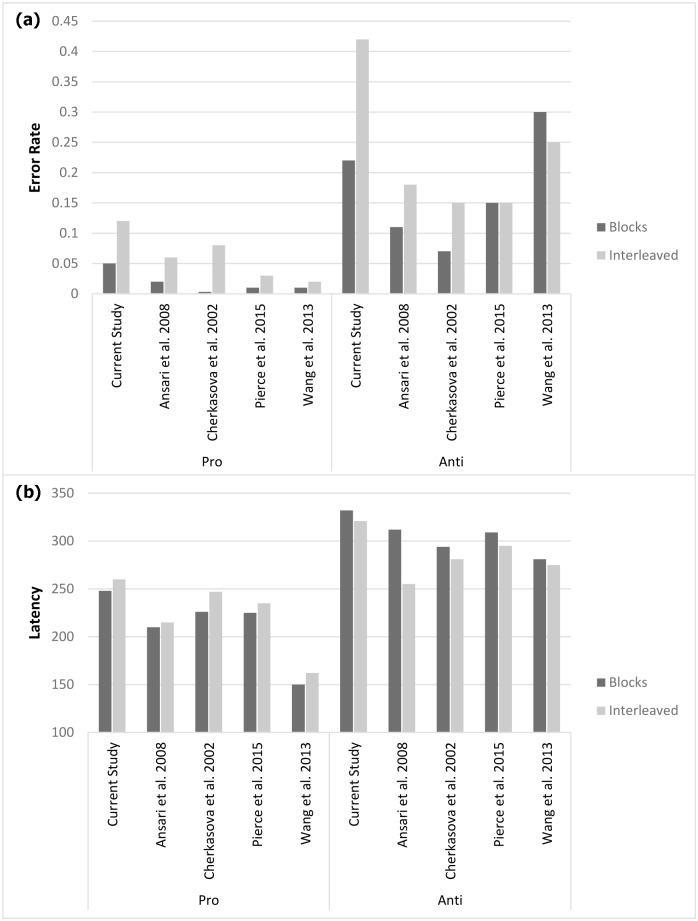
Summary of (a) error rate and (b) latency data extracted from previous studies. When precise values were not provided we estimated values from graphs. Since Cherkasova et al. [29] did not include collapsed data for "interleaved" condition we used data from "switch" condition. In Wang et al. [40] we sampled the "young" group. In Ansari et al. [28] we averaged data from high and low anxious individuals (since both represent a standard normal sample of college students).

Whether cognitive inhibitory control or increased cognitive load accounts for the current results, it should not obscure our primary point–that method of administration is a critical factor in the design of pro- and anti-saccade experiments and in the evaluation and comparison between such studies. Much of the observed results in our study are consistent with data extracted from previous studies [28–30]. These studies were not interested in the effects of administration method per se, but have all used a central-trial-cue PAT similar to our study and included some approximate variation of "block" and "interleaved" trials. These have found either pro or anti-saccade error rates to be larger in interleaved compared to block administration and/or a larger anti-saccade cost in block mode. A summary of results culled from previous papers compared to the current study is presented in Fig 6.

The summary presented in Fig 6 shows an analogous trend between our results and previous studies. Across all studies, interleaved mode seems to increase pro-saccade errors while having a marginally incremental, or insignificant, effect on latency.

**Fig 7:**
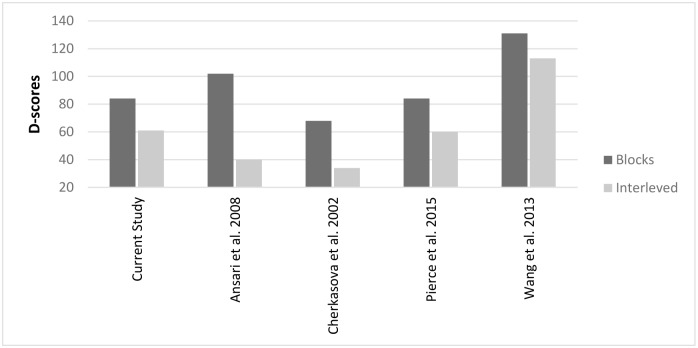
Summary of latency anti-saccade cost (computed as *D = Anti-Pro*) extracted from previous studies.

Anti-saccade latencies seemed to marginally, or insignificantly, decrease (with the exception of Ansari et al. who showed a substantial decrease). Anti-saccade error rates data was more diverse, showing a significant increase in three studies, no effect in Pierce et al. [30] and an opposite decrease in Wang et al. [40]. Fig 7 presents a summary of anti-saccade latency cost, computed as *D = Anti-Pro*. It clearly shows that block administration produce larger anti-saccade latency costs.

Thus, when designing a PAT protocol, it is crucial to consider the impact that test administration has on the outcome variables of saccadic measures. If error rate differences are the main objective, it is advised to use an interleaved mode which produces the largest differences. If, however, latency differences are the main focus, block administration yields larger differences.

One prominent discrepancy between our results and those summarized above is the relatively high anti-saccade error rates. Similar error rates are not very uncommon and have been previously documented in healthy participants (e.g. [22], [37]). In fact, a successful antisaccade generation is an effortful task, and may even reach to up to a 70% error rate in schizophrenic patients [8] making it a distinct feature in psychopathology assessment. The relatively high error rate in our study are due to our broad definition of erroneous saccades: labeling lack of eye movements as "errors" and including them in analysis, while the studies summarized above excluded such trials from further analysis. Indeed, follow up analysis showed that excluding these trials from further analysis did not change the pattern of results other than an overall 6% constant decrease in error rates across all conditions.

Results also showed that the Posner-like task exhibited analogous results to PAT only in the cued condition. Pro-saccades had both shorter latencies and shorter RT's compared to anti-saccades. However, unlike saccades, manual responses to the second target were not sensitive to administration mode. Analysis of the discrimination task also showed a facilitation effect for pro-saccades and an IOR effect for anti-saccades: In successful pro-saccade trials, faster recognition was observed when the second target appeared at the cued compared to the uncued location. Conversely, in anti-saccade trials the effect was reversed and the cued location recognition RT's was actually slower than the uncued location. These results can be understood in terms of overt attention costs: in pro trials both overt and covert attention transpose to the cued location–facilitating RT's upon target appearance. In contrast, in anti trials covert attention is directed towards the cued location and overt attention towards the uncued location. Thus, facilitated RT's at the uncued location can indicate supremacy of overt attention (engaging the uncued location). Alternatively, it is also possible that during the 600ms time window covert attention once directed to the cued location wanders back towards the engaged overt attention location exhibiting IOR for the cued location.

Indeed, IOR has been widely reported to appear at long stimulus onset asynchronys (SOAs)- when the interval between the first cue and second target exceeds ~300 ms, depending upon task [41]. However, the effect disappears when there is a temporal overlap between cue and target [42]. Our study adapted and modified a "target-target" paradigm (e.g. [31], [43]) with an SOA of 600ms and temporal overlap. This differs from the original Posner tasks where gaze remained centrally fixated [2] and should be interpreted accordingly. In addition, unlike common target-target paradigms we did not require participants to move their eyes back to the center before the second target appeared. Our task thus presents a slightly different way to measure the effects of orienting covert and overt attention on discrimination RT's. Future research is also needed in order to evaluate whether the recognition task had an effect on our results through a possible mediation of an overall cognitive load.

A limitation of the current study is the relatively narrow age range of participants. Indeed, our study, and several others [24], [44–45], have studied only young adults. This is important because age was shown to correlate with saccade performance [46]. For instance, it was shown that compared to young adults, normally aging elders displayed slower latencies and increased errors in anti-saccades and memory guided saccades [47], and slower pro-saccade [48]. Generally, it is assumed that the effects of aging are more prominent for anti-saccades, which tends to gradually improve from childhood until early 20's, plateaus for about 10 years, and then declines with aging [49]. The reported decline in anti-saccade performance suggests that the current results should also be relevant for in older adults, but that would need to be tested in future research.

## Conclusions

The present study shows that method of administration is a critical factor in the design of pro- and anti-saccade experiments. Block administration produce larger latency anti-saccade cost. Interleaved mode produce larger difference in error rates, in part by hindering the pro-saccade reflexive advantage, suggesting pro-saccades are not entirely reflexive. In contrast, Posner costs were not affected by mode of administration and showed similar results to PAT only at cued locations.

## Supporting information

S1 Table Subject's accuracy and mean reaction times. (XLSX)
